# Unlocking radiotherapy access: the critical role of structural quality and radiation therapists in Saudi Arabia

**DOI:** 10.3389/fonc.2026.1802072

**Published:** 2026-04-13

**Authors:** Zaheeda Mulla, Wsam Ghandourh, Hussain Almerdhemah, Norah Albishi, Ahmed Kattan, Amal Al Qahtani, Zainab Mansoor Alsaihaty, Ghada Aldosary

**Affiliations:** 1 Department of Oncology, King Faisal Specialist Hospital and Research Centre-Jeddah, Jeddah, Saudi Arabia; 2 Department of Health Care Management, Umm Al-Qura University, Jeddah, Saudi Arabia; 3 Department of Oncology, Prince Sultan Military Hospital, Riyadh, Saudi Arabia; 4 Department of Oncology, King Faisal Specialist Hospital and Research Centre-Riyadh, Riyadh, Saudi Arabia; 5 Department of Oncology, Johns Hopkins Aramco Healthcare, Dharan, Saudi Arabia; 6 Department of Oncology, King Fahad Specialist Hospital, Dammam, Saudi Arabia; 7 Department of Radiation Oncology, Ministry of National Guard Health Affairs, Riyadh, Saudi Arabia

**Keywords:** access to cancer care, Kingdom of Saudi Arabia, quality indicators, radiotherapy

## Abstract

**Background/objective:**

Access to radiotherapy (RT) remains a challenge even in high-income countries such as the Kingdom of Saudi Arabia (KSA). This study aims to examine how key structural indicators such as staffing and equipment availability, distribution, and capacity affect access to care and the quality of RT services in Saudi Arabia.

**Methods:**

A mixed-methods design using a descriptive cross-sectional approach was employed. A survey was distributed electronically to supervisors and managers of 20 RT units in the KSA between September 24 and October 23, 2025. The qualitative phase explored the perspectives of radiation therapists (RTTs) and medical physicists, whereas the quantitative phase examined variables such as staff numbers and distribution, patient volumes, equipment availability, treatment waiting times, and overall facility capacity between January and December 2024.

**Results:**

The survey response rate was 85% (n =20). Public facilities treated the highest caseloads but had fewer linear accelerators per patient, higher clinician workloads, and longer waiting times, with radical treatment often exceeding 14 days and, in some cases, extending beyond 42 days. The mixed centers had the highest machine capacity and advanced technologies. Training opportunities were limited, particularly in the public sector, where most departments rated staffing and operational efficiency inadequate. Equipment procurement presented a challenge with 65% of centers (majority in the public sector), expressing tendering delays severely or critically affected their ability to provide care.

**Conclusion:**

These findings support the development of a strategic framework to strengthen RT capacity and reduce sector-based disparities. Outsourcing initiatives among different facility types have already been initiated. Ultimately, these results may support policy development and resource planning to promote more equitable cancer care delivery in the country.

## 1 Introduction

Radiation therapy (RT) is a vital component of cancer treatment and is indicated for nearly 50% of cancer patients (1). Access to timely and high-quality RT is critical for effective cancer care. In the Kingdom of Saudi Arabia (KSA), rising cancer incidence rates have increased the demand for RT services, placing pressure on existing infrastructure and workforce capacity (2, 3). Structural quality indicators, such as equipment availability, facility readiness, staffing ratios, and workforce training, play a central role in determining the accessibility and efficacy of these services. Staffing adequacy, measured as the ratio of staff-to-patient volume or equipment units, serves as a key quality indicator (4). The overall quality of RT depends on the availability of well-trained staff (radiation oncologists, radiation therapists and medical physicists, and oncology nurses) and teamwork (4, 5). Radiation therapists (RTTs) play a key role in planning and delivering radiotherapy treatments, collaborating closely with oncologists, medical physicists, and nurses as part of the interdisciplinary teams in RT departments (6).

As frontline professionals, RTTs’ expertise directly affects patient outcome, treatment safety, and service efficiency. Understanding how structural quality factors and workforce capacity intersect is vital for addressing RT access gaps across KSA’s healthcare system (7). The shortage of trained RTTs remains a significant challenge in the Saudi healthcare context and may limit equitable access to RT for cancer patients (8). Furthermore, it is important to recognize that a relative surplus of capacity in one city does not necessarily alleviate the shortages in other regions.

Access to advanced and well-maintained equipment, including linear accelerators, brachytherapy units, treatment planning systems, and quality assurance tools, is another crucial indicator (9, 10). The Italian Working Group General Indicators (IWGI) link infrastructure quality to the efficiency of high-energy unit (HEU) utilization (11).

In addition, in the era of image guidance, it is crucial to ensure that RT equipment can deliver high-quality IGRT treatments. To address this, Gabriele et al. (12) introduced a quality indicator in 2016 to assess equipment adequacy for RT, termed the RT Equipment Quality Ratio, which is described in the methodology section.

Infrastructure as a quality indicator is closely associated with another structural measure, commonly referred to as “Treatment Delay” or “Waiting Time,” which reflects the total number of days from a patient’s RT referral to the start of treatment. According to the treatment intent, Cionini et al. (11) established benchmark standards for this indicator as <30 days for curative or radical treatment and <10 days for palliative or symptomatic treatment.

This study investigates the structural quality indicators that influence RT access in the KSA. By evaluating structural indicators related to the RTT and physicist workforce, this study will offer evidence-based guidance for enhancing workforce distribution and training, and for improving equipment allocation to advance access and quality of RT services in the KSA.

## 2 Materials and methods

### 2.1 Design

This study employed a mixed-methods design using a descriptive cross-sectional approach to provide a comprehensive understanding of how RTT/Medical Physicists’ workforce and equipment availability influence access to RT services in the KSA. A survey adapted from *Ramashia* et al. (2025), which used the International Atomic Energy Agency (IAEA) quality indicators framework (13, 14), was designed to collect both quantitative and qualitative data from public, private, mixed (public and private), and military radiotherapy (RT) centers.

### 2.2 Population and sampling

The survey was distributed electronically to the RTT chief/supervisors of 20 RT units in the KSA between September 24 and October 23, 2025. Although the International Atomic Energy Agency (IAEA) Directory of Radiotherapy Centres (DIRAC) reports approximately 16 centers in Saudi Arabia, national listings indicate that additional centers have recently become operational or may not yet be reflected in the DIRAC database. Therefore, the present survey targeted all 20 identified centers in order to achieve national coverage of RT service providers. The RTT chief’s/supervisor’s phone number was retrieved via the Saudi Arabian Radiotherapy Society (SART) member directory and contacted by the authors via “WhatsApp” (whatsapp.com). The communication included an invitation letter outlining the survey objectives and a link to the anonymous questionnaire that participants accessed after providing informed consent.

### 2.3 Ethical considerations

Prior to conducting this study, ethical approval was obtained from the King Faisal Specialist Hospital and Research Centre Institutional Review Board (IRB) in Jeddah, KSA (IRB Study Number:2251525).

### 2.4 Data collection

The quantitative phase was retrospective, drawing on existing data sources that examined variables such as staff numbers and distribution, patient volumes, equipment-to-staff ratios, equipment availability, and service utilization metrics such as treatment capacity, waiting times, machine downtime, and regional service coverage, from January to December 2024. Responses were provided by scoring using a Likert scale and by answering multiple-choice items. Respondents rated resource adequacy based on their professional judgement regarding whether existing infrastructure, staffing levels, and operational resources were sufficient to meet patient demand within their departments. These ratings reflected perceived adequacy relative to departmental workload rather than predefined national thresholds.

The qualitative data were derived from open-ended responses embedded within a structured, self-administered online questionnaire that mirrored a semi-structured interview format to explore the perspectives and experiences of the chief/supervisor RTT, with a section dedicated to medical physicists. The qualitative part focused on “What are the perceived challenges in RT?”. The open-ended questions were designed to elicit detailed narrative responses regarding infrastructure limitations, procurement barriers, staffing challenges, operational challenges, patient access, and health equity concerns.

#### 2.4.1 High-energy units utilization ratio

To assess the efficiency and potential overuse of equipment, the high-energy unit utilization ratio measures how many patients are treated per machine (e.g., linear accelerator) per year, where a high ratio equals efficient use *or* overburdened equipment and a low ratio equals underutilization *or* excess capacity. The IWGI (11) defines this indicator as:


$$\begin{array}{l} \text{Use of High}-\text{Energy Units }=\\ \left(\text{Total number of patients treated in one year}\right)/\left(\text{Number of HEUs}\right) \end{array}$$

#### 2.4.2 Staff workload ratio

This evaluates the number of patients that each RTT is responsible for annually. The staff workload ratio assesses whether current staffing levels are adequate, compares different sectors, and can be used as a benchmark against international standards (e.g., IAEA recommendations as outlined in *IAEA Human Health Reports No. 13* (2015); ESTRO guidelines (15). The staff workload ratio is defined as:


Workload =(Total number of patients treated in one year)/(Number of RTT staff)

#### 2.4.3 RT equipment quality ratio

To assess the adequacy of RT equipment, to determine whether it is possible to deliver high-quality IGRT treatments, Gabriele et al. (12) defines RT Equipment Quality Ratio as follows:

Adequacy of equipment for RT = (Number of IMRT/IGRT machines)/(Total number of RT machines).

By integrating both quantitative and qualitative data during the interpretation phase, this study aimed to validate and enrich the findings and provide a comprehensive assessment of how structural indicators such as the availability of RT equipment and the RTT workforce are associated with variations in access to RT services. This approach is especially well suited to the complex healthcare landscape of the KSA, where disparities in workforce distribution, equipment availability, and sociocultural factors uniquely shape healthcare delivery.

### 2.5 Statistical analysis

Data were entered into Microsoft Excel and analyzed using SPSS Version 22. Descriptive statistics, including frequencies, medians, and percentages, were used to calculate key RT quality indicators such as staff-to-equipment-to-patient ratios and average patient wait times. Analyses were conducted for the overall sample and separately according to facility type (public, private, and military facilities). Qualitative responses to open-ended questions were analyzed using a thematic analysis approach to explore the challenges and barriers to RT access, as perceived by Chief/Supervisor RTTs. Two authors independently reviewed the responses and conducted initial coding to identify recurring concepts related to infrastructure limitations, staffing challenges, procurement barriers, training opportunities, and operational constraints. Codes were subsequently grouped into broader themes through iterative discussion among the research team to ensure consistency and agreement in interpretation.

## 3 Results

Seventeen of 20 (85%) radiotherapy centers responded to the survey and were included in the final analysis, representing the majority of operational radiotherapy services nationwide. Of these 17 centers, there were nine public, five private, two mixed (treating both public and private patients) and one military center.

### 3.1 Service volume and machine utilization

Public and mixed centers treated the greatest number of patients at a mean of 911 and 2,141 patients/year, respectively, compared with a mean of 254 patients/year treated at private centers (**Table 1**). The largest facility (mixed) treated approximately 3,000 patients annually, while the smallest facility (private) treated approximately 70 patients annually (**Table 2**).

**Table 1:** Descriptive statistics of public, private and mixed radiotherapy facilities.

Variable	Public facilities (n = 9)				Private facilities (n = 5)				Mixed facilities (n = 2)			
	Mean	Mode	SD	Range	Mean	Mode	SD	Range	Mean	Mode	SD	Range
Patients/Year	911.11	1200	436.36	300-1500	254	na	143.34	70-450	2141.5	na	1212.17	1283-3000
LINACs	2.1	2	0.33	2-3	1.2	1	0.45	1-2	5	na	2.83	3-7
CT-Sim	1	1	0.5	0-2	1	1	0	1	1.5	na	.71	1-2
Brachytherapy	.67	1	.5	0-1	0	na	0	0	1	1	0	1-1
MRI-Sim	.33	na	.5	0-1	0	na	0	0	.5	na	.71	0-1
PET-CT	0.33	na	.5	0-1	.4	na	.55	0-1	.5	na	.71	0-1
RTT	8.78	9	4.53	3-17	3	3	0	3-3	22.5	na	10.61	15-30
MP	8	10	3	4-13	2.6	2	1.52	1-5	10	10	0	10-10

SD, Standard deviation; LINAC, Linear accelerator, CT-Sim, Computed tomography simulation, MRI-Sim, Magnetic resonance imaging simulation, PET-CT, Positron emission tomography; RTT, Radiation therapist; MP, Medical physicist.

**Table 2:** Linear accelerators (LINACs) utilization rates in different radiotherapy centers.

Center ID	Center type	Patients/year	Number of LINACs	LINACs utilization rate (patients/LINAC)
1	Public	850	2 (1)^2^	425 (850)
2	Public	1500	2	750
3	Public	450	2	225
4	Public	300	2	150
5	Public	1300	2	650
6	Public	1200	2	600
7	Public	400	2	200
8	Public	1000	3	333.3
9	Public	1200	3	400
10	Private	300	1	300
11	Private	70	1	70
12	Private	200	2	100
13	Private	450	1	450
14	Private	250	1	250
15	Mixed^1^	1283	3	427.7
16	Mixed^1^	3000	7	428.6
17	Military	700	2	350

^1^Accepting both public and private patients, ^2^number of Linear accelerators currently in use.

Machine availability differed markedly across sectors. Public centers operated a mean of 2.1 linear accelerators (LINACs), compared with 1.2 in private centers and 5 in mixed centers (**Table 2**). When normalized per 1,000 patients, private centers demonstrated the greatest machine density (4.72 LINACs/1,000 patients) followed by military (2.86), public (2.44), and mixed facilities (2.33) (**Table 3**). LINAC utilization rates reflected this disparity with public centers treating between 150–750 patients per LINAC annually compared to 428 patient/LINAC in mixed centers and around 70-300/LINAC in private centers.

**Table 3:** Normalized linear accelerator (LINAC) availability in different sectors (LINACs per 1,000 patients).

Sector	Number of LINACs	Patients/Year	LINACs/1,000 patients
Public	20 (19)^2^	8200	2.44 (2.32)
Private	6	1270	4.72
Mixed^1^	10	4283	2.33
Military	2	700	2.86

^1^Accepting both public and private patients, ^2^number of linear accelerates currently in use.

### 3.2 Availability of simulation and treatment technologies

CT simulation units were present in nearly all facilities, although one public center reported operating without one unit. Advanced imaging technologies, such as MRI simulation and 4D-CT, were concentrated in private and mixed centers, whereas several public facilities lacked one or more of these capabilities despite delivering the majority of treatments (**Table 4**).

**Table 4:** Number of linear accelerators (LINACs) and CT simulation machines in different radiotherapy centers. .

Center ID	Center Type	LINAC’S	LINACs used for IMRT/IGRT	Adequacy of equipment for RT	CT	PET-CT	4D-CT	MRI-Sim
1	Public	2 (1)^2^	2	1	1	⊗	1	1
2	Public	2	2	1	1	1	⊗	⊗
3	Public	2	2	1	1	1	⊗	⊗
4	Public	2	2	1	1	⊗	⊗	⊗
5	Public	2	2	1	1	⊗	⊗	⊗
6	Public	2	2	1	2	⊗	2	1
7	Public	2	2	1	1	⊗	⊗	⊗
8	Public	3	3	1	⊗	1	⊗	1
9	Public	3	3	1	1	⊗	⊗	⊗
10	Private	1	1	1	1	1	1	⊗
11	Private	1	1	1	1	1	⊗	⊗
12	Private	2	2	1	1	⊗	1	⊗
13	Private	1	1	1	1	⊗	⊗	⊗
14	Private	1	1	1	1	⊗	⊗	⊗
15	Mixed^1^	3	3	1	1	⊗	⊗	⊗
16	Mixed^1^	7	7	1	2	1	2	1
17	Military	2	2	1	1	⊗	1	⊗

^1^Accepting both public and private patients, ^2^number of Linacs currently in use, IMRT: Intensity-modulated radiation therapy, IGRT: Image-guided radiation therapy, CT: Computed tomography, PET-CT: Positron emission tomography, 4D-CT: Four-dimensional computed tomography, MRI-Sim: Magnetic resonance imaging simulation, ⊗ Not available.

All centers provided 3D conformal radiotherapy, IMRT, and VMAT, and were fully equipped to deliver IMRT/IGRT treatments. However, the availability of advanced treatments varied. TBI was available in seven of the 17 centers (41%), none of which were private facilities. Brachytherapy was available in only a subset of facilities (53%), predominantly in public and mixed settings, whereas procedures such as Total Skin Electron Therapy (TSET) and Intraoperative Radiation Therapy (IORT) were rarely reported (**Table 5**).

**Table 5:** Availability of different radiotherapy technologies in the facilities.

Center ID	Center type	3D-CRT	IMRT	VMAT	SBRT	TBI	Tomotherapy	DIBH	Water bath	TSET	IORT	Brachytherapy
1	Public	✓	✓	✓	✓	⊗	⊗	✓	✓	⊗	✓	✓
2	Public	✓	✓	✓	⊗	⊗	⊗	✓	⊗	⊗	⊗	⊗
3	Public	✓	✓	✓	✓	✓	⊗	⊗	✓	⊗	⊗	✓
4	Public	✓	✓	✓	✓	⊗	⊗	✓	⊗	⊗	⊗	⊗
5	Public	✓	✓	✓	✓	✓	⊗	✓	⊗	⊗	⊗	✓
6	Public	✓	✓	✓	✓	✓	⊗	✓	⊗	⊗	⊗	✓
7	Public	✓	✓	✓	⊗	⊗	⊗	✓	⊗	⊗	⊗	⊗
8	Public	✓	✓	✓	✓	⊗	⊗	⊗	⊗	⊗	✓	✓
9	Public	✓	✓	✓	✓	✓	✓	✓	⊗	⊗	⊗	✓
10	Private	✓	✓	✓	✓	⊗	⊗	✓	⊗	⊗	⊗	⊗
11	Private	✓	✓	✓	✓	⊗	⊗	✓	⊗	⊗	⊗	⊗
12	Private	✓	✓	✓	✓	⊗	⊗	✓	⊗	⊗	⊗	⊗
13	Private	✓	✓	✓	✓	⊗	⊗	✓	⊗	⊗	⊗	⊗
14	Private	✓	✓	✓	✓	⊗	⊗	✓	⊗	⊗	⊗	⊗
15	Mixed^1^	✓	✓	✓	✓	✓	✓	✓	✓	⊗	⊗	✓
16	Mixed^1^	✓	✓	✓	✓	✓	✓	✓	⊗	✓	✓	✓
17	Military	✓	✓	✓	✓	✓	⊗	✓	⊗	⊗	⊗	✓

^1^Accepting both public and private patients, 3D-CRT, Three-dimensional conformal radiation therapy; IMRT, Intensity-modulated radiation therapy; VMAT, Volumetric modulated arc therapy; SBRT, Stereotactic body radiation therapy; TBI, Total body irradiation; DIBH, Deep inspiration breath hold; ABC, Active breathing control; TSET, Total skin electron therapy; IORT, Intraoperative radiation therapy, ✓Available, ⊗ Not available.

### 3.3 Workforce capacity and workload

Workforce data showed substantial variation among the sectors. In public centers, the number of radiation therapists varied between 3 and 17 while the number of medical physicists ranged between 4-13. As such, the workload (patients/year) ranged between 60–500 with a mean of 133 patients/year for radiation therapists and between 60–250 with a mean of 118 patients/year for medical physicists (**Table 6**).

**Table 6:** Workload ratio for staff in different radiotherapy facilities.

Center ID	Center type	Patients/year	Number RTTs	Number of MP	RTT workload (patients/year)^2^	MP workload (patients/year)^3^
1	Public	850	9	10	94.4	85
2	Public	1500	3	6	500	250
3	Public	450	7	7	64.3	64.3
4	Public	300	5	5	60	60
5	Public	1300	14	7	92.9	185.7
6	Public	1200	11	10	109.1	120
7	Public	400	4	4	100	100
8	Public	1000	9	13	111.1	76.9
9	Public	1200	17	10	70.6	120
10	Private	300	3	1	100	300
11	Private	70	3	2	23.3	35
12	Private	200	3	3	66.7	66.7
13	Private	450	3	2	150	225
14	Private	250	3	5	83.3	50
15	Mixed^1^	1283	15	10	85.5	128.3
16	Mixed^1^	3000	30	10	100	300
17	Military	700	13	4	53.8	175

RTT, Radiation therapist; MP, Medical physicist, ^1^Accepting both public and private patients, ^2^Recommended: 100-150, ^3^Recommended: 300-400.

In private centers, the number of radiation therapists averaged around three, and the number of medical physicists ranged between 1-5. The workload ranged between 23-150 (mean = 84) for therapists and 35-300 (mean= 135) for medical physicists. In mixed centers, there were 22 radiation therapists and 10 medical physicists, with an average workload of 93 and 214 patient/year for therapists and medical physicists, respectively (**Table 6**).

### 3.4 Training and professional development

**Figure 1:**
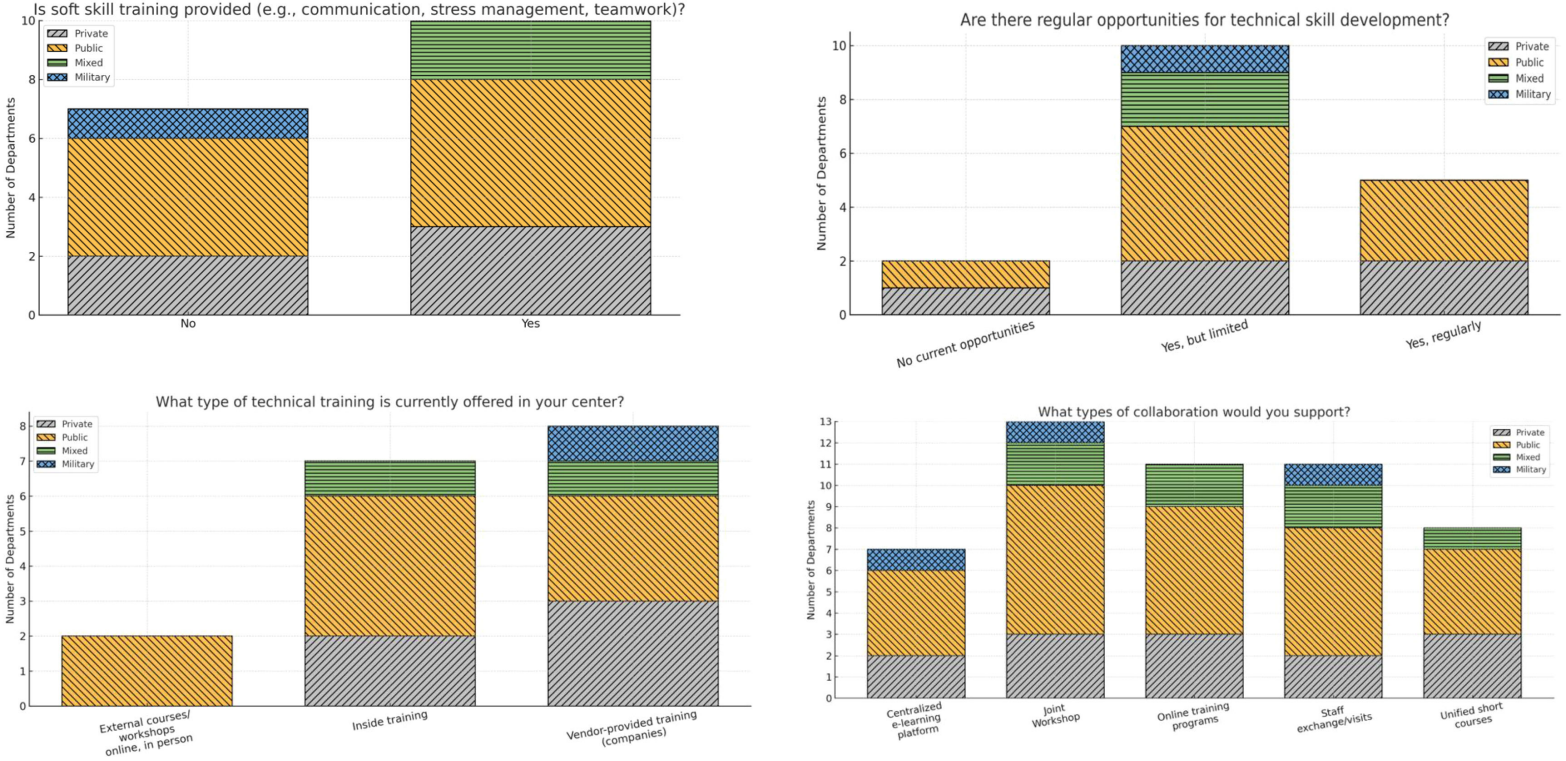
Training provided within departments from different sectors.

Training and professional development opportunities were limited, with 59% of departments reporting only limited technical skills training (related to radiotherapy technologies and procedures such as treatment planning systems, image-guided radiotherapy, treatment delivery techniques, and quality assurance) and almost half indicating no structured soft skills training (referred to non-technical competencies including communication, teamwork, leadership, patient experience and interaction, and multidisciplinary collaboration); these gaps were most pronounced in the public sector. Regular technical training (structured training activities occurring at least quarterly or more frequently) occurred in 29.4% (5/17) of the centers, 58.8% (10/17) reported limited opportunities, and 11.8% (2/17) reported none. Soft-skill training was absent in 41.2% (7/17) of centers. Public centers accounted for the majority of sites that lacked structured training pathways. The preferred collaboration strategies included staff exchanges (47.1%), centralized online training (41.2%), and joint workshops (35.3%) (**Figure 1**).

The majority of the available training was provided by vendors (47%), followed by internal training (41%), while external courses/workshops were limited to only 12%. The majority of the survey participants expressed a strong desire for training opportunities, particularly in the form of joint workshops, staff exchange visits, and online training programs (**Figure 1**).

### 3.5 Waiting times

**Figure 2:**
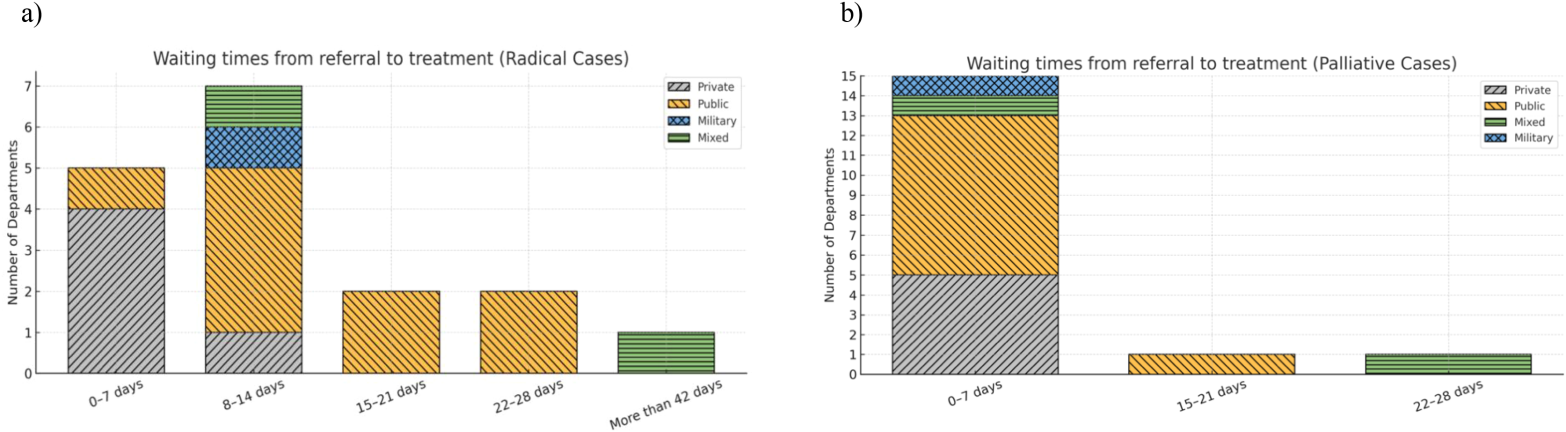
Waiting times for patients from radiotherapy referral to start of treatment for Radical cases **(a)** and Palliative cases **(b)**.

Most departments reported starting palliative radiotherapy within 0–7 days of referral, with only a small number of public and mixed centers treating palliative patients after more than 2 weeks. For radical radiotherapy, private departments were more likely to initiate treatment within 0–14 days, whereas public departments more often reported waits extending beyond three weeks, including almost all instances of delays longer than 28–42 days (**Figure 2**).

### 3.6 Perceived resource adequacy

**Figure 3:**
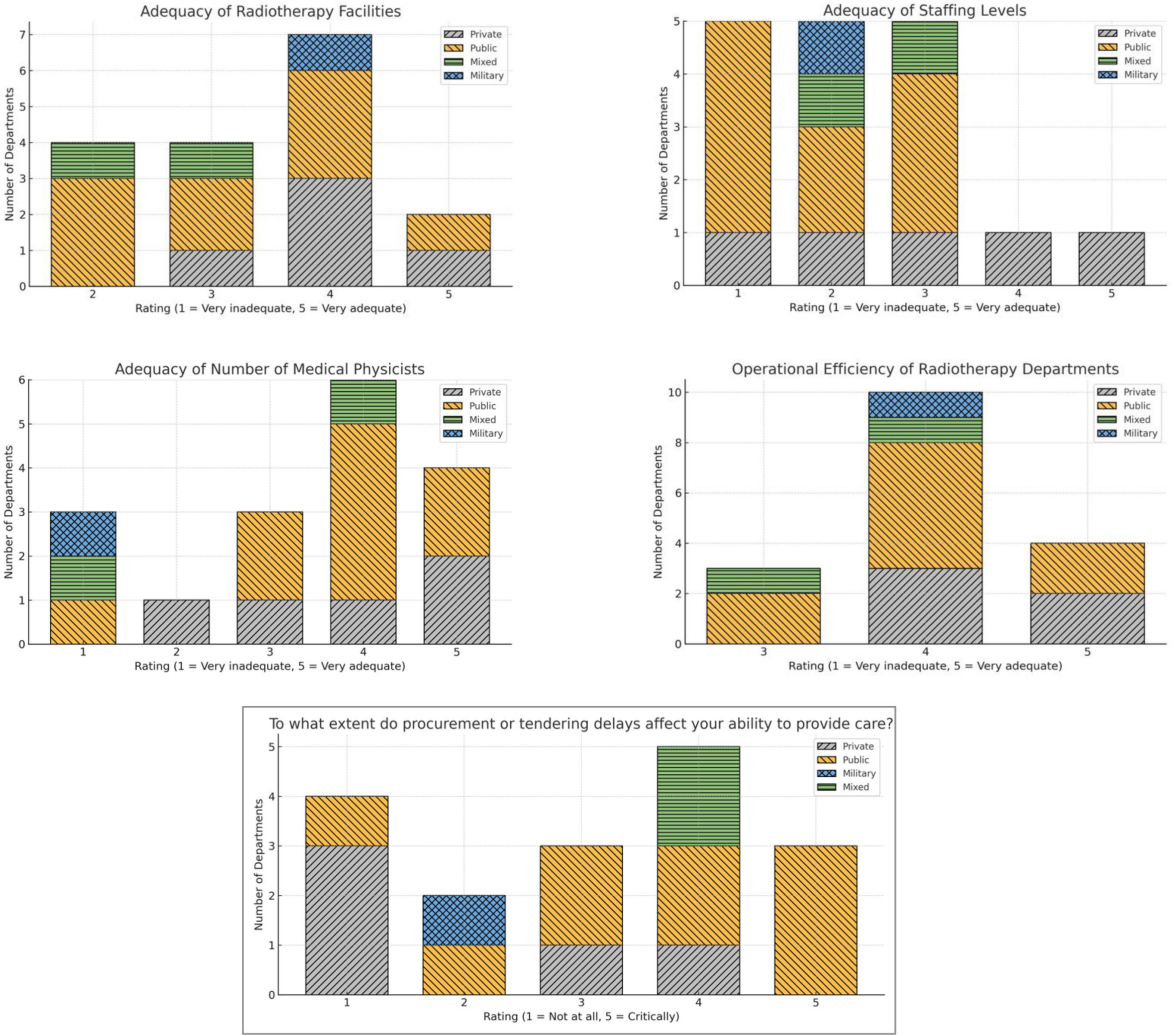
Perceived ratings of resource adequacy and effects of procurement delays in different sectors.

Perceptions of resource adequacy and operational efficiency differed notably between public and private radiotherapy departments. Overall, most departments rated facilities, staffing, and efficiency in the mid-range of the 5−point scale, suggesting that resources were generally sufficient to meet demand, but rarely viewed as optimal. With respect to radiotherapy facilities, both public and private departments most frequently selected ratings of 3 and 4, indicating moderate to good adequacy in meeting patient demands. However, private departments contributed a greater proportion of ratings at the upper end of the scale, whereas public departments were more often represented among the midrange scores (**Figure 3**).

Staffing levels showed a less favorable pattern, particularly in public institutions. Ratings were clustered between 1 and 3, with public departments reporting very low adequacy levels more frequently. Private departments also reported constraints but were more likely than public centers to select intermediate ratings. With regard to medical physics staff, the numbers were generally higher than those of overall staffing, but sectoral differences persisted. Private departments were more commonly associated with ratings of 4 and 5, while public departments demonstrated a wider spread of responses, including more mid-range and lower ratings (**Figure 3**).

Finally, operational efficiency ratings clustered around intermediate-to-high levels, with ten departments rating efficiency at Level 4. Notably, none of the departments reported critically low operational efficiency (ratings 1-2), indicating that functional workflows are maintained despite resource constraints in specific areas (**Figure 3**).

### 3.7 Procurement and tendering

Procurement and tendering delays were reported by five departments as having a severe impact (rating 4), representing the largest response category. An additional three departments reported critically severe impacts (rating 5), indicating that delays fundamentally compromised their ability to deliver radiotherapy services (**Figure 3**). Only four departments indicated minimal impact from procurement delays (rating 1), three of which were private.

Qualitative comments reinforced these findings, describing procurement processes as “slow,” “bureaucratic,” and “complex,” with delays attributed to multi-layered approval systems. Additionally, lack of radiotherapy-specific expertise within purchasing departments led to issues such as receiving incomplete or inappropriate items due to poor understanding of clinical requirements. Several respondents reported that procurement delays directly affected treatment schedules, particularly when spare parts or radiotherapy-specific consumables were involved. Conversely, a minority described their processes as either efficient or improved over time.

## 4 Discussion

Despite being a high-income country, the KSA has only 1.15 linear accelerators per million people, which is below international benchmarks, resulting in significant regional disparities in RT services (2). In a previous study, we explored uneven access to radiotherapy in the KSA, with the northern and southern regions lacking linear accelerators (2). This national mixed-methods assessment provides one of the most comprehensive evaluations to date of radiotherapy (RT) structural capacity, workforce distribution, and service access inequities across the KSA. The findings of this study reveal significant variability in staffing adequacy, equipment distribution, training opportunities, and treatment timeliness across different facility types. Despite substantial national investments in cancer services, these disparities suggest that gaps in structural quality may be associated with differences in access to high-quality RT care. Structural indicators such as workforce availability, equipment capacity, and operational efficiency are widely recognized as key determinants of RT quality, safety, and patient outcomes in both high- and middle-income settings (2, 4, 5).

A prominent finding of this study is the substantial variation in patient volume and machine utilization across facility types. Public centers delivered the majority of treatments, mixed centers reported greater volumes, and private centers managed comparatively lower caseloads. This imbalance may reflect systemic referral pathways, insurance limitations, and patient preferences that appear to direct a larger proportion of patients toward the public sector. Furthermore, public centers averaged just over two LINACs each, private centers averaged slightly more than one, and mixed centers reported the highest capacity at five. These patterns suggest that some public centers may be operating under higher service pressure, exceeding the internationally recommended thresholds of ~350–450 patients per LINAC, as cited by the IAEA and ESTRO guidelines (11–13). In contrast, some private facilities operated below these benchmarks, suggesting variability in machine utilization across sectors and mixed centers are within the recommended threshold. Such integration is increasingly recommended in international radiotherapy planning models, particularly where demand–capacity mismatches exist (8, 9).

Advanced imaging and treatment technologies, which are critical for achieving contemporary standards of precision radiotherapy, are disproportionately concentrated in mixed and private centers. While all surveyed centers provided core modalities, access to advanced simulation imaging technologies (**Table 4**), and TBI remained limited (**Table 5**). The unequal distribution of advanced equipment in many public-sector centers may contribute to disparities in treatment quality across facility types, and may influence clinical outcomes, the adoption of modern protocols and workflow efficiency. Similar disparities have been documented globally, particularly in rapidly developing RT systems, where expansion occurs more quickly than workforce readiness or procurement alignment (10).

Workforce capacity has emerged as another area with pronounced disparity. Several centers operate with staffing levels that exceed internationally recommended workload thresholds for both radiation therapists and medical physicists (**Table 6**) (13–16), in keeping with perceptions of resource adequacy being less than optimal in most centers. Staff shortages arising from remuneration disparities and challenging working conditions appear to represent an important structural challenge affecting the timely and effective delivery of radiotherapy care (9). Participants in this study described clear and significant salary gaps between centers in the same geographic areas, noting that differences in annual raises and contract renewal bonuses between public and private sectors adversely affect the equitable distribution of experienced staff. Among the challenges related to staff shortages that were experienced, was “Staff burn out and absenteeism,” “Delay in patient appointments,” “Working longer hours, frustration, and higher demands for vacations,” “lack of quality due to improper rest.” These findings highlight the need for a coordinated national workforce strategy that addresses systemic staffing pressure in public, private and mixed centers while accounting for geographical, cultural, and sector-specific constraints (13, 14, 16). Gender-based mobility limitations may also influence the geographical distribution of female RTTs across regions (17).

Training and professional development capacities are limited in many centers, particularly in the public sector. The absence of structured technical and soft skills training in a substantial proportion of departments reflects a heavy reliance on vendor-based education and limited access to external courses. However, although vendor-led training represents an important source of technical education, it may not substitute for structured national continuing professional development (CPD) programs that ensure standardized competencies and long-term professional development across institutions. Given the technological complexity of radiotherapy, gaps in training may pose risks to service quality and could limit the safe implementation of advanced techniques (16, 18). International evidence consistently links inadequate training with workflow inefficiencies and treatment errors (4, 5, 19).

The observed waiting times for RT treatment may also be associated with underlying structural differences between facilities. While most centers initiated palliative RT within acceptable timeframes, as indicated by Cionini et al. (11), delays for radical RT were common in public and mixed centers, which may reflect underlying structural differences in machine capacity, staffing levels, and operational workflows. Prolonged waiting times have been shown to reduce tumor control, worsen survival, and increase psychological distress and are considered one of the most critical structural quality indicators in radiotherapy (2, 11). Thus, waiting time inefficiencies observed in public centers may indicate broader systemic issues, including insufficient machine capacity, inadequate staffing, scheduling bottlenecks, and operational challenges.

Procurement and tendering delays were reported as significant operational challenges affecting the continuity of treatment. Public centers, in particular, reported severe or critically severe impacts from slow bureaucratic procurement processes. Streamlining procurement workflows, engaging RT professionals in technical purchasing decisions, and establishing rapid response mechanisms with a dedicated budget for essential components are important steps toward improving operational efficiency (9).

According to the IAEA Directory of Radiotherapy Centres (DIRAC), Saudi Arabia operates approximately 16 radiotherapy centers with around 45 megavoltage (MV) machines, which remains below international benchmarks for population coverage (20). In comparison, Egypt operates approximately 71 radiotherapy centers with 124 MV machines, while Morocco maintains around 30 centers equipped with 49 MV machines, despite lower national income levels (20). Within the Gulf Cooperation Council, variation is also evident, with the United Arab Emirates operating 10 radiotherapy centers with 19 MV machines, Kuwait maintaining two centers with five MV machines, and Qatar providing radiotherapy through a single center equipped with four MV machines (20). Jordan, although smaller in population, operates five centers with 20 MV machines and benefits from more favorable staffing ratios and lower workloads per radiation therapist, aligning with the structural quality indicators outlined by Ramashia et al. and the IAEA (9, 13, 14). Morocco’s “Plan Cancer 2020–2029” expansion to more than 80 linear accelerators and over 52 centers by 2025 exemplifies rapid capacity growth supported by structured RTT training pathways (21).

In contrast, several high-volume Saudi centers continue to operate with insufficient staffing relative to workload, despite the influx of new graduates. The need for coordinated national planning to better align academic training with workforce needs, particularly given that radiation therapy education programs are limited to only two or three universities located in the central region of Saudi Arabia, contributing to regional concentration of workforce supply and demand (17).

Collectively, these comparisons suggest that the challenges observed in KSA may be related not only to technological capacity but also to structural and organizational factors, including uneven workforce distribution, salary and benefit disparities between the public and private sectors, sectoral fragmentation, and limited public–private coordination. Therefore, strengthening national governance frameworks and adopting integrated planning models similar to those implemented in Jordan and Morocco may enhance equity and service resilience (2, 11, 13, 14). Several recommendations to overcome uneven workforce challenges have been suggested in the following quotes: “Offer relocation packages, guaranteed safe housing, and transport allowances for remote posts,” “Implement flexible scheduling and onsite/nearby childcare or childcare stipends,” “Provide remote/onsite CPD and rotational programs so staff can rotate into tertiary centers for training.” “Use targeted recruitment campaigns and financial top-ups for hard-to-fill regions.” “Create clear career pathways and faster licensing/recognition for qualified RTTs to improve retention.”

Despite these challenges, this study showed notable strengths. Operational efficiency ratings were generally moderate to high, suggesting that many departments maintain functional workflows despite resource pressure. The presence of advanced technologies, with all centers delivering high-quality IGRT treatments, also positions the country for rapid expansion of high-precision RT services, as workforce and procurement barriers are addressed. Moreover, the expressed desire among RTTs and physicists for enhanced training and collaboration reflects a committed and motivated workforce, aligning with Atun et al.’s call for new global approaches to radiotherapy training, including core curricula and innovative learning methods (19).

### 4.1 Policy implications

The findings of this study highlight several policy priorities for strengthening radiotherapy capacity in the Kingdom of Saudi Arabia. First, the higher patient volumes and machine utilization observed in public-sector facilities suggest the need for targeted workforce distribution strategies and additional staffing support in high-burden centers. Second, the presence of underutilized capacity in some private facilities indicates potential opportunities for structured public–private partnerships to redistribute patient load and reduce waiting times in the public sector. Third, the procurement delays reported by several departments highlight the importance of streamlining tendering processes, improving technical expertise within procurement units, and establishing faster mechanisms for the acquisition of essential radiotherapy equipment and spare parts. National policy makers should establish equipment procurement policies that could address current delays for radical treatment in public centers. Finally, the training gaps identified across many centers suggest the need for coordinated national CPD programs to support the safe implementation of advanced radiotherapy technologies and maintain workforce competencies, rather than relying solely on vendor-based education. Together, these measures could support more efficient resource allocation and improve equitable access to radiotherapy services across the country.

### 4.2 Limitations

First, qualitative insights were derived from written responses to open-ended survey questions rather than recorded interviews, which may limit the depth of contextual detail captured. Second, the survey focused primarily on radiation therapists and medical physicists and did not include radiation oncologists, as previous international data indicate that radiation oncologist staffing levels are comparatively adequate (20). Finally, several centers were unable to provide complete operational data, such as machine downtime ratios, which limited the ability to assess the machine downtime ratio as a quality indicator.

## 5 Conclusion

This study suggests that RT access in the KSA may be influenced by multiple structural factors, including workforce capacity, equipment allocation, training infrastructure, and procurement processes. Disparities between public and private sectors may undermine equitable access to radiotherapy. Addressing these interlinked challenges may help support the delivery of more timely, high-quality, and equitable radiotherapy services nationwide. Outsourcing initiatives between different facility types have already been initiated. This study’s findings provide timely evidence that may inform outsourcing initiatives, strategic planning, policy development, and investment decisions aimed at strengthening national RT capacity and reducing disparities across sectors and regions.

## Data Availability

The raw data supporting the conclusions of this article will be made available by the authors, without undue reservation.
